# Health System Factors Influencing the Integration of Pre-Exposure Prophylaxis into Antenatal and Postnatal Clinic Services in Cape Town, South Africa

**DOI:** 10.9745/GHSP-D-24-00166

**Published:** 2024-12-20

**Authors:** Lara Court, Aurelie Nelson, Reghana Taliep, Sarah Schoetz Dean, Rufaro Mvududu, Lucia Knight, Kathryn Dovel, Thomas Coates, Landon Myer, Dvora L. Joseph Davey

**Affiliations:** a Division of Social and Behavioural Sciences, School of Public Health, University of Cape Town, Cape Town, South Africa.; b Division of Epidemiology and Biostatistics, School of Public Health, University of Cape Town, Cape Town, South Africa.; c Western Cape Department of Health and Wellness, Metro Health Services, Cape Town, South Africa.; d Division of Infectious Diseases, Geffen School of Medicine, University of California Los Angeles, Los Angeles, CA, USA.; e School of Public Health, University of the Western Cape, Bellville, South Africa.

## Abstract

**Introduction:**

Oral pre-exposure prophylaxis (PrEP) is an effective and safe option to prevent HIV acquisition and vertical HIV transmission in pregnant and breastfeeding women. Understanding health system factors influencing the integration of PrEP into care for pregnant and breastfeeding women is key to increasing access. We explored managers’ and health care workers’ (HCWs) experiences with integrating PrEP into antenatal care and postnatal care services in primary health care clinics in Cape Town, South Africa.

**Methods:**

This exploratory qualitative study used codebook thematic analysis, where HCWs were purposively, heterogeneously sampled from an implementation science study. Semistructured individual interviews were conducted with 9 managerial-level staff, and 3 focus group discussions were conducted with HCWs (nurses, midwives, and HIV counselors) providing PrEP (6–7 HCWs per group) between November 2022 and January 2023 (N=28). Interview guides covered health system facilitators, barriers, and recommendations. The Health Systems Dynamics framework guided data analysis and presentation of results.

**Results:**

PrEP integration into antenatal care services was described as acceptable and feasible; however, changes to HIV testing policy and indicators in breastfeeding women are needed to integrate PrEP into postnatal clinics, together with identification of mother and baby as a dyad in visits. Results showed that supportive policies facilitated wider, simplified PrEP provision. The availability and accessibility of prescribing nurses and lay HIV counselors, PrEP (both within facilities and in communities), and information about PrEP for implementers and pregnant and breastfeeding women will be pivotal to facilitating integration.

**Conclusion:**

Facilitators for PrEP integration include task-shifting PrEP education and identification of women for PrEP initiation to HIV counselors, changes to national guidelines defining who can prescribe PrEP, revision and integration of PrEP training for HCWs, community-level interventions for PrEP demand creation and stigma reduction, and provision of differentiated PrEP delivery options.

## Abstract (precis)

Integrating PrEP for pregnant and breastfeeding women into antenatal and postnatal clinic services requires supportive PrEP prescription and HIV testing policies, improved availability of trained nurses and counselors, together with simplified access to PrEP and related information, both in clinics and communities.

## Teaser

**Key Findings:**

- Effective use of oral pre-exposure prophylaxis (PrEP) by pregnant and breastfeeding women is limited despite its potential to reduce HIV incidence and vertical HIV transmission.
- To understand health system barriers and facilitators to integrate PrEP into antenatal and postnatal clinic care, we explored the perspectives of managers, midwives, nurses, and counselors providing PrEP services in South Africa.
- Barriers included shortages of and limited access to trained staff for PrEP delivery, coupled with limited policies to facilitate simplified PrEP provision in all clinics.
- Facilitators included prioritizing task-shifting and task-sharing of PrEP delivery, treating the mother and baby as a dyad, and increasing PrEP awareness for health care workers and clients.

**Key Implications:**

- Antenatal and postnatal PrEP delivery should be simplified by optimizing clinic visit flow, revisiting health care worker training on PrEP, and task-shifting and task-sharing to all nurses and HIV counselors, together with using community-level strategies, including HIV self-testing, differentiated PrEP delivery, and improved PrEP messaging, to facilitate awareness of PrEP and reduce stigma.
- Policymakers should consider adapting national PrEP prescription policy and policy guidelines outlining increased, pre-established HIV testing time points postpartum with comprehensive monitoring through national indicators.

## INTRODUCTION

Pregnant and breastfeeding women remain at high risk for HIV acquisition in South Africa, with effects extending beyond their health to that of their children.^1^ Behavioral factors, including limited condom use, sexually transmitted infections, and changes in physiology, such as increased vaginal inflammation during pregnancy, play a role in this increased risk.^2^^–^^5^ Antenatal HIV testing, as well as uptake of lifelong antiretroviral therapy (ART) among pregnant and breastfeeding women, has resulted in a 60% reduction in HIV vertical transmission in Eastern and Southern Africa.^6^^,^^7^ However, 43% of global incident vertical transmissions still occur in the region.^8^ Approximately one-third of vertical HIV transmissions occur postnatally during breastfeeding,^6^^,^^9^ and one-third occur following acute HIV infection among pregnant and breastfeeding women, when viral load is at its highest.^10^^,^^11^ Daily use of oral tenofovir-based pre-exposure prophylaxis (PrEP) is both safe and effective in preventing HIV acquisition among pregnant and breastfeeding women,^12^^,^^13^ with PrEP use among at-risk pregnant and breastfeeding women included in World Health Organization guidelines.^14^ Recent models in South Africa show that PrEP use among pregnant and breastfeeding women has the potential to reduce vertical transmission between 13% (conservative scenario) and 41% (optimistic scenario).^15^ In response, the South African National Department of Health adapted the national PrEP guidelines to include pregnant and breastfeeding women in 2021.^16^

Despite its potential, PrEP provision for and uptake by pregnant and breastfeeding women has been slower than expected in sub-Saharan Africa.^17^^,^^18^ Following initial international guidance,^19^ PrEP provision in sub-Saharan Africa has focused primarily on at-risk populations, specifically sex workers and adolescent girls and young women,^20^ and has often been integrated into facility- and community-based sexual and reproductive health services.^21^ This follows continued calls for provision of integrated HIV care, including HIV prevention, both in facility and community settings (e.g., primary health care services).^22^ Integration of HIV care, including prevention, has the potential to strengthen health systems, improving both program outcomes and reach of services in a cost-effective and sustainable manner while simultaneously making services more client-centered and increasing ART uptake.^23^ With the focus of PrEP provision moving onto pregnant and breastfeeding women, integration of PrEP into maternal and child health clinics has the potential to increase access to PrEP as a primary prevention tool for pregnant and breastfeeding women living without HIV.^1^^,^^17^^,^^24^

Integration of PrEP into sexual and reproductive health services has been described as feasible and acceptable according to perspectives of adolescent girls and young women and HCWs.^25^^–^^29^ However, these same populations have expressed PrEP integration and uptake as hindered by HIV-related stigma, low perceived risk for HIV infection, fear of intimate partner violence linked with inadvertent PrEP disclosure, negative HCW attitudes and beliefs, insufficient HCW knowledge of PrEP, and constraints in workforce resources.^25^^–^^30^ The limited evidence available for the integration of PrEP into health services for pregnant and breastfeeding women in sub-Saharan Africa describes it as feasible,^31^ despite uptake of PrEP by pregnant and breastfeeding women being lower than expected.^32^ Barriers to integration for pregnant and breastfeeding women are similar to those documented among adolescent girls and young women.^26^^,^^33^^–^^35^ Identification of factors enhancing integration in real-world settings is scarce, with those available including task-shifting (from doctors to nurses or nurses to lay counselors), optimization of clinic visit flow (i.e., fast tracking), and adequately training HCWs.^26^^,^^34^

Evidence demonstrates that when exploring integration of facility-based health services, the inclusion of HCWs’ perspectives is key to understanding barriers and facilitators to integration, as well as recommendations for interventions^26^^,^^36^ while considering the influence of all health system elements.^12^^,^^37^ This qualitative study used a health system lens to explore perspectives of HCWs overseeing and providing PrEP in an implementation science study.^32^ We aimed to understand supportive and obstructive health system factors relating to the integration and uptake of PrEP for pregnant and breastfeeding women into antenatal care (ANC) and postnatal care (PNC) clinics, together with HCW recommendations to overcome experienced barriers, allowing exploration of how to facilitate integration in this context.

We aimed to understand supportive and obstructive health system factors relating to integrating PrEP for pregnant and breastfeeding women into antenatal care and postnatal care clinics.

## METHODS

This qualitative study formed part of a larger implementation science study evaluating the integration of PrEP services into ANC and PNC services following training and mentorship of HCWs.^32^ Clinics were located in the Klipfontein and Mitchells Plain subdistricts of Cape Town, South Africa, both high-density areas with considerable antenatal HIV prevalence (10% to 35%).^38^ Participating facilities (4 City of Cape Town health clinics and 4 larger Cape Town Metro health service community health centers) offered services for pregnant and breastfeeding women, delivered either in a building or in pregnancy-specific maternity delivery units. PNC services included healthy baby check-ups at regular intervals, infant immunization, and infant measurements. These clinics followed 2019 National ART guidelines^39^ and 2021 National Department of Health PrEP guidelines,^16^ detailing PrEP prescription by ART-trained nurses only,^40^^,^^41^ defined as nurses who are either clinical nurse practitioners or have completed the Western Cape Nurse-Initiated and Managed Antiretroviral Treatment (NIMART) program.^41^

An exploratory qualitative study design^42^^,^^43^ facilitated the use of in-depth interviews (IDIs) and focus group discussions (FGDs) to understand the experiences of routine HCWs involved in PrEP delivery for pregnant and breastfeeding women after training and mentorship, conducted between November 2022 and January 2023. Participants provided written informed consent. Grocery vouchers (ZAR100, ∼US$6) were provided for participant time and efforts. Topic guides covered health system facilitators and barriers and recommendations pertaining to PrEP integration and were informed by initial findings from the initial Cape Town-based PrEP in Pregnancy cohort study,^1^ relevant literature,^24^^,^^44^^,^^45^ and the Theoretical Framework of Acceptability.^46^ Member checking was built into topic guides as a rigor strategy. Experiences in overseeing integration of PrEP in facilities were explored through 9 IDIs^47^ with purposely sampled, relevant program coordinators and managers overseeing 4 ANC and PNC clinic sections from 4 of the 8 participating clinics that had received training and mentorship for at least 3 months. To understand the collective experience of PrEP provision embedded within standard service delivery in ANC and PNC settings,^32^ 3 separate FGDs were held with 6–7 purposively sampled HCWs per discussion. Participants in FGDs strategically included active ANC and PNC nurses or lay HIV counselors from participating clinics who had received training and mentorship for at least 3 months.

**FIGURE 1:**
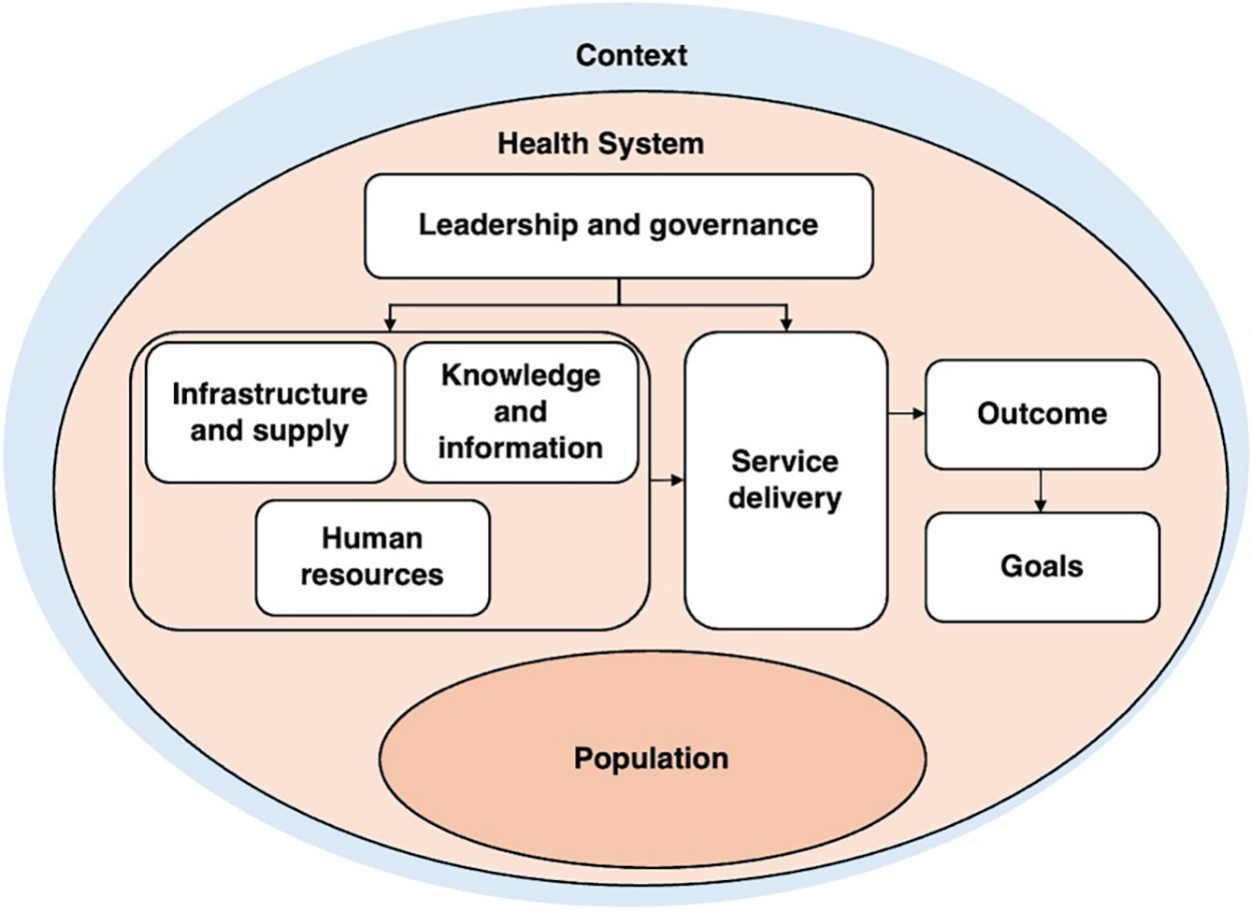
Health Systems Dynamics Framework Source: Olmen et al.^48^

**FIGURE 2:**
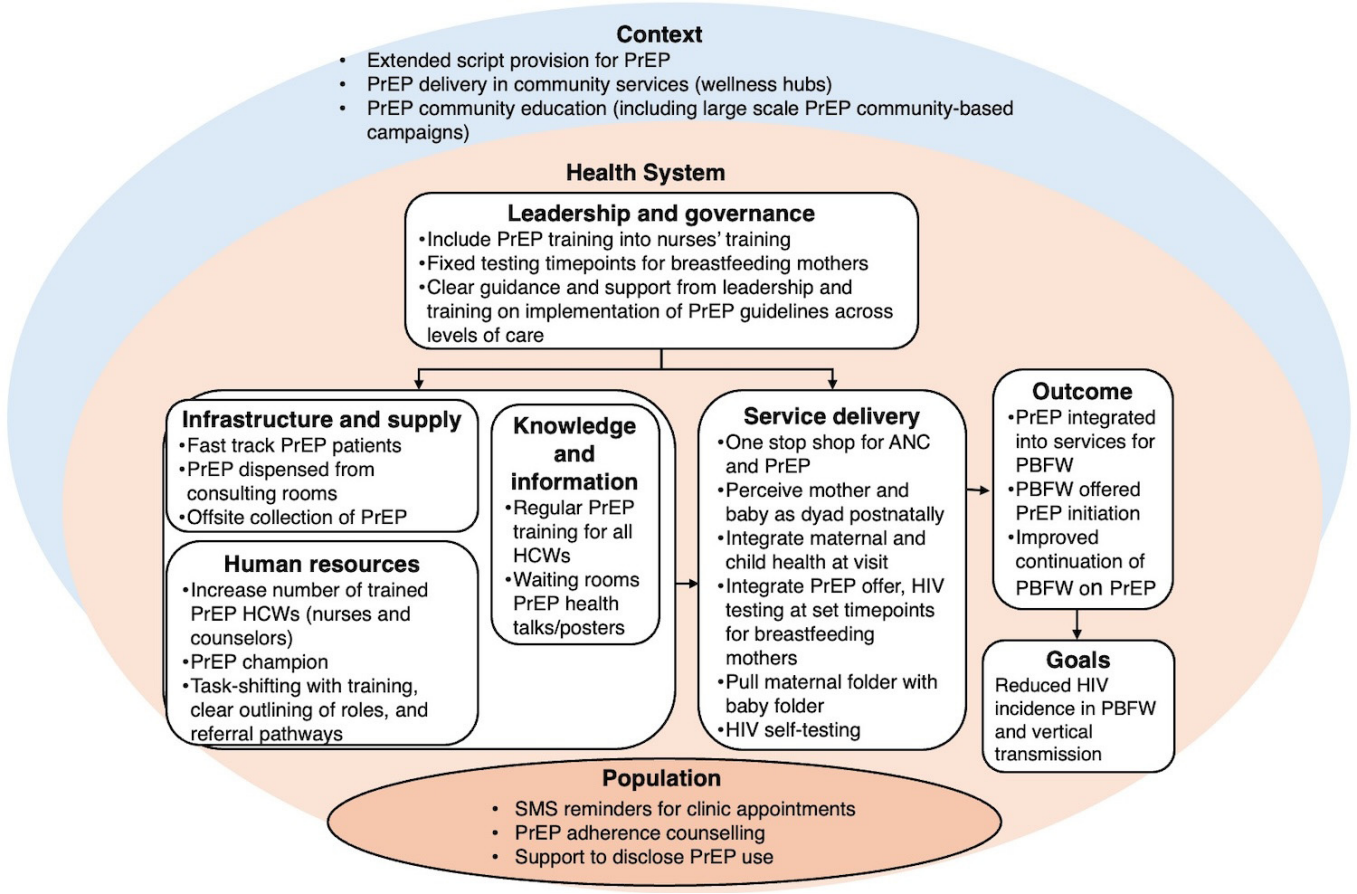
Health Systems Dynamics Framework Adapted to HCWs’ Recommendations and Perceived Facilitators of Integration of PrEP into ANC and PNC Services Abbreviations: ANC, antenatal care; HCW, health care worker; PBFW, pregnant and breastfeeding women; PNC, postnatal care; PrEP, pre-exposure prophylaxis; SMS, short message service.

The Health Systems Dynamics (HSD) framework guided our conceptualization of the health system for analysis. The framework includes 10 dynamic and interrelated elements (Figure 1). It informed our understanding of how integration of PrEP into existing facility-based services for pregnant and breastfeeding women could affect or be influenced by other health system elements, local context, and the population of pregnant and breastfeeding women. This further facilitated the development of health system recommendations for future programs according to influential elements (Figure 2).^48^ Sections of the Patient-Centered Access to Health Care framework provided a mechanism to explore how contextual factors and population-specific characteristics impact accessibility of integrated PrEP services, specifically pregnant and breastfeeding women’s ability to perceive, seek, reach, engage, and pay for services.^49^

Codebook thematic analysis was used.^50^ Data was co-coded by 3 researchers (LC, AN, who ran the larger study, and RT), facilitated by computer-assisted qualitative data analysis software Dedoose (version 9.0).^51^ A codebook was developed (LC) following familiarization, using broad categories based on the interview guide topics, results from the larger study,^32^ and the HSD framework.^52^ A set of transcripts was co-coded, deductively and inductively initially, to further build out the codebook, after which each researcher coded a subset of transcripts independently, which were then reviewed (LC). Informed by the interpretivist epistemological stance, researchers were actively involved in making meaning^53^^–^^55^ using memos, annotations, and regular debriefing meetings to ensure rigor and a journal to ensure reflexivity.^53^^–^^56^ Upon completion of coding, the research team (including international collaborators and the field team) peer reviewed thematic analysis results. Subthemes and themes were further analyzed, refined, and categorized using the HSD framework, presented as such in the results section. Specific subthemes related to HCW acceptability will be reported elsewhere (RT). The Critical Appraisal Skills Programme checklist for qualitative research was followed,^57^ together with rigor strategies guided by Lincoln and Guba’s principles of credibility, confirmability, dependability, and transferability.^58^

### Ethical Approval

Ethical approval was attained from University of Cape Town Human Research Ethics Committee (ref 117/2022), the Western Cape Health Research Committee (ref WC_202206_003), and City of Cape Town Health Research Committee (ref 8148).

## RESULTS

IDIs were conducted with 9 program coordinators and managers, including 6 operations managers from 3 Cape Town Metro health service clinics and 1 City of Cape Town health clinic manager. Managers had a mean of 14 years of experience in their current position. A total of 19 HCWs, including nurses, midwives, and lay HIV counselors, involved in delivering PrEP at 4 of the participating clinics took part in 3 FGDs, described by total and those trained to prescribe ART and PrEP (Table). All HCWs were female, except 2 managerial staff, a common trend in nursing in South Africa.^59^

We report HCWs’ experiences of health system factors perceived as obstructive and conducive to both the integration of PrEP in ANC and PNC clinic services as well as access to PrEP by pregnant and breastfeeding women in these services, together with provider recommendations to overcome experienced barriers. These are reported according to the HSD framework, specifically under interrelated themes of (1) leadership and governance, (2) human resources, (3) knowledge and information, (4) infrastructure and supplies, (5) service delivery, (6) contextual factors influencing access, and (7) population characteristics influencing access. In the final 2 themes, access to integrated PrEP services was informed by the Patient-Centered Access to Health Care framework, specifically pregnant and breastfeeding women’s ability to perceive, seek, reach, engage, and pay for services. Results are summarized in Figure 2.

### Leadership and Governance

Participants described how the national policy requiring nurses to be ART trained to prescribe and dispense PrEP^40^ reduced the number of available prescribers, hindering pregnant and breastfeeding women’s initiation and continuation on PrEP. They also felt that the ART training’s (NIMART) current format was long, complex, and technology-heavy, making it challenging for nurses to complete.

Participants described how the national policy requiring nurses to be ART trained to prescribe and dispense PrEP reduced the number of available prescribers.

*Because even though the NIMART structure has changed, it’s still a process… And some of those processes involve technology and doing some online work…Our doctors and nurses are already short [limited] in facilities, freeing them up to do that training… So, it could take up to 3, 4 months.* —Program coordinator

Delayed access to or application of current PrEP policy guidelines inhibited integration. Inconsistent implementation of PrEP policies in clinics and across levels of care was described as impacting PrEP service continuity, particularly the follow-up of pregnant and breastfeeding women who needed referral while pregnant or in the transitional period from pregnancy to postpartum. Current policies around HIV testing for breastfeeding mothers also limited integration due to misalignment between baby’s immunization schedule and recommended HIV testing intervals, resulting in testing not occurring timeously and missed opportunities for offering PrEP.

*…Mothers who breastfeed [are supposed] to be tested [for HIV] every 3 months. The problem is that it… doesn’t fit into, well, the baby visits. The baby doesn’t come every 3 months.… We test for HIV when baby comes again.* —Program coordinator

To facilitate integration, HCWs recommended increased HIV testing of breastfeeding mothers by aligning postnatal testing schedules with standard baby visits in national policy. They described how greater access to trained PrEP prescribers could be achieved by collaboration between local governance structures (i.e., Provincial and National Department of Health, Pharmacy Council, and South African Nursing Council) to revise the current ART-training structure and integrate PrEP training into other HCW trainings (i.e., formal nursing qualifications or accredited short-courses), together with adaption of national policy to allow non-NIMART trained nurses to prescribe PrEP.

*The problem is… only the NIMART trained sister can prescribe and dispense that medication [PrEP]…Who is going to be running services while the nurse is in training?… The registered nurse and mid-wife know exactly what to do, just a small opportunity, for them to be given access to prescribe and dispense.* — Program coordinator

Participants also requested support and troubleshooting from Provincial and National Departments of Health following new PrEP policy development, specifically to overcome challenges in implementing monitoring and evaluation procedures, and to gain clarity on guidelines. Clear and well-communicated guidelines were described as facilitating integration.

*The [previous] guideline wasn’t that clear… But the current guideline gives more clarity on how to proceed with work up and questioning of the pregnant and breastfeeding women at risk. And how to monitor them clinically. So that has made a difference.* — Program coordinator

### Human Resources

A lack of available and appropriately trained HCWs presented as a significant challenge to integration. Counselor and health promoter shortages limited provision of PrEP education and HIV testing in some clinics, inhibiting demand creation, initiation, and continuation on PrEP, as these were difficult to include in clients’ short consultations with nurses. Limited numbers of ART-trained nurses in sections delivering services to pregnant and breastfeeding women was a primary barrier to PrEP integration. Counselors and nurses unable to prescribe PrEP described trouble initiating women when ART-trained nurses were either “too busy,” located in other sections of the clinic, or absent entirely, as a nurse explained.

*With our facility … so we had a slow start [in offering PrEP] because I’m not NIMART trained. So, I rely on [a] clinical nurse practitioner. The clinical nurse practitioner assigned to me that day was … on leave. So, I had struggles with somebody prescribing. Then I had to go to [another clinic section], to the doctor there. And the doctor feels like this is not my [his] job… I need a prescriber because what’s the point of offering a service and there isn’t somebody to help me initiate.* —Nurse, FGD

The inclusion of PrEP into existing services in the context of staff shortages created a logistical burden for a few HCWs, making it difficult to prioritize PrEP among other duties. This was particularly relevant for staff working in PNC clinics, where providing PrEP to breastfeeding women increased their workload and required a shift in focus from the baby to the mother during consultations. A counselor noted that following the delivery of PrEP education to a mother, PNC clinic nursing staff would sometimes forget to include the subsequent offer to initiate or continue PrEP.

*There are more nurses trained on PrEP, but they do not introduce it. Like, I am alone. I can’t go to all the mommies to introduce. So, those [nurses] that see the babies after birth, they don’t introduce the PrEP to those mommies, and they are breastfeeding.* —HIV lay counselor, FGD

The workload distribution between those able to prescribe PrEP and others was perceived as unequal. ART-trained nurses felt overburdened and “distracted” by regular requests to “sign off” on PrEP prescriptions. In parallel, counselors described their workload as increased as compared to others because of more HIV tests required to monitor PrEP use and a larger number of clients to educate. Managers voiced how this could result in friction between staff.

*It impacted the workload a lot. Even more so on the side of the counselors. You find that sometimes there are conflicts because of that increasing workload. And then, as the manager you have to intervene. You have to say, “let’s use these ways of trying to work it out.”* —Operations manager

In addition to ensuring sufficient numbers of trained HCWs for PrEP delivery, some HCWs endorsed employing dedicated PrEP staff or having a specific HCW act as a “PrEP champion.” Clarification of roles of HCWs providing PrEP before integration was encouraged, together with task-shifting strategies, such as shifting PrEP prescription to all trained nurses and midwives to provide a “one-stop shop” for PrEP initiation. The presence of available and appropriately trained staff for PrEP delivery was a key facilitator of integration, and facilities “flowed” when they had access to PrEP-trained HCWs and PrEP prescribers in sections working with pregnant and breastfeeding women. Together with role designation, making HIV counselors responsible for HIV testing, PrEP education, and counseling also facilitated PrEP delivery. Clear referral processes between ART-trained nurses and those educating pregnant and breastfeeding women on PrEP were found helpful in the context of staff shortages and policy challenges.

*The sister [nurse] that is doing the babies immunizations for the breastfeeding mum, she is referring them just opposite to the sister that is NIMART trained… it [PrEP delivery] is working well, because they are doing it.* —Operations manager

### Knowledge and Information

HCWs further described how a lack of PrEP knowledge inhibited integration. Nurses described situations where HCWs in other facilities or clinic sections were not aware of the use or safety of PrEP in HIV prevention or confused PrEP with ART for HIV treatment. This resulted in PrEP not being offered to pregnant and breastfeeding women in consultations or in a more serious situation, such as when a woman referred to a hospital from the clinic was prescribed the incorrect medication.

Nurses described situations where HCWs in other facilities or clinic sections were not aware of PrEP’s use in HIV prevention or confused PrEP with ART.

*… there was also a confusion in [hospital name] when our patients went over [there], because they [the hospital] had not started with PrEP. They didn’t know what it this. They thought they were on ART. So, we rather write PrEP, not TDF [Tenofovir disoproxil fumarate] … Don’t write that. ‘Cause now, that side, they think it’s antiretroviral…They’re only sending us the guidelines now. But we already know now.* —Nurse, FGD

To overcome this, in-service training of all HCWs and facility staff, regardless of direct roles in PrEP delivery, was recommended to increase PrEP awareness (what it is and its availability) and emphasize PrEP education as a collective staff responsibility to support task-sharing. Participants also endorsed specific training for PNC clinic nurses on the risks of vertical transmission to encourage nurses’ perception that both the mother and child are clients during consultations and should promote the offer of frequent HIV testing and PrEP to breastfeeding women.

*Breastfeeding… we need to make sure that the moms that come to the clinic get tested for HIV… And I think often times you are like, “Ah she was negative in pregnancy” … This job is done. She seroconverts, she breastfeeds, and the baby is [HIV] positive… So maybe it is also a training issue that staff aren’t aware of it. And, if there is more awareness of that fact, they are more likely to test.* —Program coordinator

Other strategies were recommended to increase PrEP information for clients to stimulate further demand for PrEP, including discussing PrEP during health talks by counselors or nurses in the waiting room, which was found to facilitate integration, and having physical resources (e.g., posters and pamphlets) to remind clients and staff about the availability and effectiveness of PrEP in prevention of HIV in pregnant and breastfeeding women without HIV.

### Infrastructure and Supplies

Long lines and queuing to collect PrEP at the clinic pharmacy were described as a possible barrier to pregnant and breastfeeding women continuing PrEP. Recommended strategies to overcome delays in collecting PrEP at the facility included having a designated space for PrEP collections or a separate pharmacy queue for PrEP. Nurses referenced strategies used to speed up medication collection, such as direct dispensing during consultations, among others, as they found that these strategies facilitated integration.

*For me I give a trauma slip … I’m bypassing [the] system … So, they go straight to the window. Their folder gets created. And within 5 minutes they are back in. My issue is pharmacy … They’re taking too long. I fast-track these people and they tell me it’s not warranted. Then people get fed-up…* —Nurse, FGD

Although it poses possible challenges, specifically regarding monitoring,^60^ off-site PrEP collection through existing ART (or PrEP delivery) clubs, electronic lockers, or chronic medication dispensing units used in South Africa was also recommended by participants.

*So, I think looking at our external spaces and I think we’d need to … make use of the e-lockers more. The e-lockers have been underutilized… for patients even on chronic medicine. So why not add PrEP?* —Program coordinator

### Service Delivery

HCWs described how providing PrEP as a part of ANC facility-based services was largely feasible, as women were visiting the clinic regularly and HIV testing was routine, but that it could be inhibited by general service delivery challenges (i.e., timing of blood test collection, lack of or faulty appointment systems, queues to access facility). To mitigate these service-related barriers, nurses recommended that PrEP delivery be fully integrated into ANC consultations rather than requiring navigating additional sections of the clinic.

*I think in pregnancy, there is not too many [barriers], I don’t think so. Because you go to the clinic, you get tested. Maybe a barrier is that you get sent and you have to see the counselor. So, it’s not a one-stop shop. So, just, uhm, makes your visitor longer and you have to queue again.* —Program coordinator

HCWs described how breastfeeding women were more difficult to identify for PrEP initiation and follow-up through facility-based PNC clinics. Mothers who did not attend the infant visit themselves (e.g., another family member or person attended with the baby) presented as a specific challenge, and because those who attended PNC clinic consultations were also not always perceived as the “client,” they were not offered PrEP or given their own folder. In addition to making sure that mothers’ clinical care folders were available during PNC clinic consultations, HIV self-testing was recommended as a possible strategy to provide breastfeeding women with an option to HIV test off-site, described by 1 HCW as particularly applicable in scenarios when mothers were absent from baby wellness or child immunization visits.

*Because then when the baby is there, you give it to the mom … she can actually test if she is [there], if the mom is not there, if a caregiver brings a baby, uhm, you can ensure that mom can test at home, with [HIV] self-screening.* — Program coordinator, 9 years in role

Integration of PrEP services into off-site health facilities (i.e., wellness hubs or mobile clinics) was also recommended in the context of increasing pregnant and breastfeeding women’s ability to reach follow-up services and continue with PrEP. HCWs encouraged collaboration between community actors within this and mentioned places and organizations where pregnant and breastfeeding women were referred, described health committees and education structures as a few key targets, and noted that this recommendation may indirectly promote overall community knowledge and awareness of PrEP.

*Yes! … Community-based delivery… the very people that are facilitating the community-based delivery are going to be the same people that are going to spread the gospel of the fact that there is a PrEP program… So that the facility doesn’t become the only place they hear about PrEP…* —Operations manager

### Contextual Factors Influencing Access

Contextual factors were perceived to play a role in the accessibility and uptake of integrated PrEP services for pregnant and breastfeeding women. Firstly, HCWs noted how, in addition to access challenges common to other populations (i.e., inconvenient clinic hours, facility or mobile clinics not within walking distance, and lack of finances for transportation), a unique barrier to PrEP service access and continuation among pregnant and breastfeeding women was geographical migration, either during delivery or the postpartum period.

*I think the only thing now is when they go out of the province. Because you’re only allowed to give them 3 [month prescriptions]. So, I don’t know how it is going to happen. They do [stay at their rural homes]. Those that are not working they do stay … So now they are going to miss [their PrEP].* —Nurse, FGD

Nurses and counselors also described how a lack of awareness and understanding of PrEP in local communities impacted pregnant and breastfeeding women’s ability to perceive and engage in PrEP services. Nurses detailed how associations of PrEP with an unfaithful partner or “cheating” and misconceptions that PrEP use required life-long continuation or increased one’s risk for HIV presented as barriers to uptake, as well as how a lack of permission from family members or partners to use PrEP made some pregnant and breastfeeding women apprehensive to engage in use. This, together with confusion between PrEP versus ART and PrEP eligibility, meant that pregnant and breastfeeding women were hesitant to start PrEP or disclose their PrEP use out of fear of HIV-related stigma. Fear of a possible HIV diagnosis was also thought to impact use, particularly on breastfeeding women’s motivation for HIV testing, a prerequisite for PrEP initiation and continuation.

*Firstly, I think perhaps it is fear, fear regarding the outcome of the test … They [breastfeeding women] don’t give you a problem, but, when it comes to having to get an HIV test, then somehow, somewhere, they are afraid.* —Operations manager

### Population Characteristics Influencing Accessibility of Integrated Pre-Exposure Prophylaxis Services

HCWs felt that pregnant and breastfeeding women experienced similar barriers or facilitators to engaging with PrEP to those in other populations.^45^ They described possible unique challenges, such as how taking oral PrEP daily may be taxing in the context of physical symptoms of pregnancy (i.e., frequent nausea or vomiting), especially when pill fatigue or PrEP side effects were experienced in addition to these; how pregnant and breastfeeding women’s motivation to initiate PrEP may be influenced by a lack of trust in PrEP’s safety for their baby; and how motivation to engage with PrEP circumstantially shifts over time, with a drop off after birth.

*Once baby is there, you don’t want to remember a tablet every day. You’re going to forget … babies are busy, and it is a new adjustment, especially for first time mom … So a tablet that they must take just for in case, is not going to be a priority.* —Operations manager

To overcome these, HCWs recommended individual-level strategies to promote pregnant and breastfeeding women’s ability to engage with PrEP, including using short message service reminders for appointments and missed appointment follow-ups. Assistance regarding PrEP disclosure was also recommended to promote family support, ideally facilitating continuation and reducing stigma around PrEP use.

HCWs recommended individual-level strategies to promote pregnant and breastfeeding women’s ability to engage with PrEP.

*Yes, you must have a support system at home. It’s also another way to destroy that stigma. A way to normalize PrEP use … So, a child will know that “my mom takes these pills at this time.” Then they will remind their mom…* —Operations manager

PrEP adherence counseling was another strategy suggested to help pregnant and breastfeeding women with “taking ownership of their health” (specifically after pregnancy), using aspects addressed in similar counseling for other chronic conditions like diabetes or hypertension. HCWs also recommended the use of long-acting PrEP methods to support adherence.

*It [long-acting PrEP] would work very well as well because… A person won’t be able to say, “I forgot to take it today” … Because with the daily oral PrEP, there are chances that the patient may forget… Even adherence [continuation], because the patient will know that they will come after 2 months, just like how they come for family planning … that is also something that would motivate them.* —Operations manager

## DISCUSSION

We described the collective experiences of South African Department of Health managers, nurses, and lay HIV counselors in integrating PrEP into existing facility-based services for pregnant and breastfeeding women following a 6-month intervention that included in-person didactic and practical PrEP training and ongoing mentorship. Our results provide context to understand the slower-than-expected uptake of PrEP and low continuation in the larger implementation science study^32^ and add to existing research^26^^,^^30^^,^^31^ to provide recommendations for future PrEP integration programs for pregnant and breastfeeding women based on real-world experiences. The primary barrier to PrEP integration included a shortage of and limited access to appropriately trained staff, as found by others integrating PrEP into female-specific services.^25^^,^^26^^,^^30^^,^^34^ Providers recommended supportive local and national policies to facilitate wider, simplified PrEP provision, together with clear communication and support from leadership and governance structures regarding PrEP implementation in facilities across different levels of care. Further recommendations and driving factors of adequate integration include the availability and accessibility of having trained HCWs for PrEP delivery, specifically prescribing nurses and lay HIV counselors; PrEP, both in facilities and in the community; and information about PrEP for implementers and pregnant and breastfeeding women through training and other resources (Box).

BOX Health Care Workers’ Recommendations to Facilitate Pre-Exposure Prophylaxis Integration in Antenatal Care and Postnatal Care Clinic Services for Pregnant and Breastfeeding Women

- Treat mother and baby as a dyad and increase maternal HIV testing to facilitate integration of pre-exposure prophylaxis (PrEP) into postnatal care clinics.
- Ensure access to PrEP prescribers through PrEP training and supportive prescription policies.
- Prioritize task-shifting and task-sharing to all nurses and HIV counselors responsible for PrEP delivery through training on PrEP with establishment of referral pathways and role clarification, while ensuring equitable work-load distribution and sufficient number of HIV counselors.
- Optimize clinic flow and use of community-level strategies to increase access to PrEP in- and outside of facilities (community-differentiated care).
- Consider interrelatedness of health system elements in intervention design.

A unique human resource challenge in this study relevant to the Western Cape was the insufficient number of ART-trained nurses to prescribe PrEP in ANC and PNC clinic settings.^61^ HCWs recommended an integrated approach between PrEP policymakers and implementers to adapt prescription guidelines and support integration. Specific suggestions included the revisiting of training policies and the structure of required NIMART training that allows PrEP prescription, as well as including PrEP training in nursing schools’ curricula or other training for nurses, echoing other HCW recommendations.^62^ Supportive policies and accessible training could also facilitate pregnant and breastfeeding women’s PrEP continuation by ensuring sufficient access to PrEP prescriptions within a range of facilities, particularly important for postpartum women who are no longer engaged in ANC,^63^ something highlighted by others to help prevent further vertical transmission.^64^^,^^65^

Nurses and counselors also endorsed task-shifting to facilitate delivery of PrEP in the context of staff shortages, together with task-sharing,^66^ wherein responsibility for PrEP delivery is shared through facility-wide training, allowing more points of contact to access PrEP. Helpful task-shifting strategies experienced included the presence of referral pathways between those involved in the PrEP delivery for pregnant and breastfeeding women in a facility, particularly between prescribing nurses and others. Clear delineation and clarification of roles were found to facilitate integration, recommended further by HCWs and other studies,^30^^,^^34^ including the creation of a “PrEP champion” or encouraging lay HIV counselors to provide PrEP education, counseling, and screening, while nurses focus on initiating pregnant and breastfeeding women onto PrEP during consultations. Task-shifting in resource-constrained African settings to increase access to HIV services and optimally use HCW resources has received much attention.^67^^,^^68^ HCW recommendations echo this strategy for delivering PrEP to pregnant and breastfeeding women, aligning with World Health Organization recommendations^69^ and recent evidence describing the possible value and acceptability of task-shifting in PrEP delivery, where nurses and community health workers have historically provided HIV services.^66^^,^^70^ Care needs to be taken to manage HCW burden by increasing the number of lay HIV counselors, effectively training HCWs for their new roles,^20^ and investigating perceptions of unequal workload distribution within roles, which HCWs reported to influence motivation to integrate PrEP. Although briefly described here, HCWs’ acceptability of integrating PrEP into ANC and PNC clinic services in the context of task-shifting processes^66^ requires further exploration.

Managers, nurses, and counselors described how integrating PrEP into facility-based ANC services was feasible, as once a woman is pregnant, she has regular engagement with facility services,^71^ and HIV testing was an accepted part of ANC, also described in other contexts.^24^ However, integrating PrEP delivery for breastfeeding women into PNC clinics was documented as challenging due to difficulties in identifying breastfeeding women eligible for PrEP. HCWs described poor implementation of HIV testing policies for breastfeeding women, the lack of specific services, and insufficient integration of HIV testing for mothers attending baby’s immunization visits as key contributors, worsened by the resistance to HIV testing by some mothers. Breastfeeding women attending PNC clinic consultations are often not considered clients and attend without personal folders, making HIV testing, PrEP initiation, and continuation of prescriptions difficult. With mothers living with HIV also endorsing not being asked about their ART adherence or viral load in their child immunization visits, it is possible that the lack of integrated maternal and child care may all contribute to postnatal vertical transmission of HIV.^72^ In ANC services, the mother and in utero baby are treated as a dyad,^74^ a perception which needs to continue with breastfeeding women as recommended at a global and national level^23^^,^^73^ to minimize risk of vertical transmission after birth. This could be addressed through continued training on postnatal vertical transmission for nurses in PNC clinics. National policy could support this by pairing fixed HIV testing dates for breastfeeding women with baby immunization visits, as well as through creating a national indicator to monitor HIV testing and results as well as PrEP uptake among breastfeeding women. Practically, HCWs recommended “pulling” mothers’ medical records at PNC clinic consultations and referring breastfeeding women after delivery for PrEP continuation support.

Integrating PrEP delivery for breastfeeding women into PNC clinics was documented as challenging due to difficulties in identifying breastfeeding women eligible for PrEP.

Although integrating PrEP into existing ANC services was described as beneficial to PrEP accessibility for pregnant women, linking PrEP delivery to facilities brought certain challenges, particularly for breastfeeding women. Echoing previous studies,^27^^,^^30^ HCWs described optimizing clinic visit flow through strategies to increase access to PrEP within facilities (i.e., specific pharmacy queues strictly for PrEP and dispensing PrEP during nurse consultations). They noted that these should be provided in addition to community-level interventions, designed in consultation with related community structures, and adapted to the needs and contextual considerations of pregnant and breastfeeding women. This included options for HIV self-testing, differentiated delivery of PrEP outside of the clinic^74^ (in particular, off-site collection of PrEP with HIV self-testing to monitor serostatus^20^), use of long-acting PrEP methods, and provision of extended scripts to support PrEP continuation for pregnant and breastfeeding women, including those relocating to other geographic areas during the peripartum period. Exploration of HCW acceptability of these interventions is underway and will be reported elsewhere. Also described in other studies,^26^^,^^30^^,^^34^ all cadres stated how PrEP messaging needed to move past “facility walls” to facilitate demand creation. To generate awareness of PrEP availability and reduce HIV stigma attached to PrEP use, participants recommended multimodal community-level campaigns should focus on dismantling myths and confusion regarding PrEP eligibility and PrEP versus ART. Existing large-scale PrEP campaigns in South Africa predated the inclusion of pregnant and breastfeeding women as a target population.^75^^,^^76^ Future campaigns would benefit from taking recent evidence into account,^20^ demonstrating possible negative effects of creating demand through focusing on “risk” in target populations.^77^^–^^79^

### Strengths and Limitations

This study has several strengths and limitations. We employed qualitative methods to explore the perspectives of HCWs who underwent training and mentorship in the larger implementation study. The training and mentorship to provide PrEP may have informed participant views and, therefore, could differ from HCWs experienced in other approaches to integrating PrEP into ANC and PNC clinic services.^24^ Further understanding of the influence of the larger study’s model of integration (i.e., integrating PrEP through training and mentorship) is recommended. Furthermore, pregnant and breastfeeding women or innovation recipients were not included as part of this analysis, but rather population barriers and facilitators were described according to the HCW’s (innovation implementors) perspectives. Perspectives of pregnant and breastfeeding women involved in the larger implementation study have been published elsewhere.^4^^,^^80^ Researcher subjectivity is inherent in qualitative methods; however, reflexivity and other rigor strategies outlined by the Critical Appraisal Skills Programme^57^ and Lincoln and Guba’s criteria^58^ were followed. While contextual findings are not generalizable outside the study setting,^81^ contextual factors and other thick descriptions have been provided to enhance transferability, together with the use of the HSD framework^81^ to extend lessons learned. IDIs and FGDs conducted in isiXhosa were translated into English, potentially resulting in a loss of original meaning. To reduce this, quality assurance checks of translation, together with peer review and discussion with the interviewer, were performed following analysis.^82^

## CONCLUSION

Managers and providers (nurses, midwives, and lay HIV counselors) described how integrating PrEP into ANC and PNC clinic services required a system-wide approach. Counselors, providers, and managers described integrating PrEP into ANC as feasible; however, they described breastfeeding women engaged in PNC clinic services as a particularly challenging population regarding identification and PrEP prescription and counseling services.

Potential facilitators of integration and recommendations to overcome barriers included targeting shortages of appropriately trained staff through policy changes to prescription guidelines, revisiting the ART-training structure, integrating PrEP into other training and task-shifting strategies, using collaborative community-level interventions to target pregnant and breastfeeding women’s awareness and understanding of PrEP to create demand for and reduce stigma around PrEP, and increasing access to PrEP in facilities and off-site through differentiated delivery options for PrEP. Using PrEP to assist in reducing HIV acquisition and vertical transmission during breastfeeding will require a specific set of additional strategies, ranging from policy adaptations to practical service delivery strategies.

**TABLE.** In-Depth Interview and Focus Group Discussion Participant Characteristics, 8 Health Clinics, Cape Town, South Africa

**Job Position**	**No.**	**Can Prescribe ART or PrEP, No. (%)**
In-depth interviews	9	
Program coordinator	2	–––
Operations/health clinic manager	7	4 (57)
Focus group discussions	3	
Midwife	4	2 (50)
Nurse	9	3 (33)
HIV lay counselor	6	––

Abbreviations: ART, antiretroviral therapy; PrEP, pre-exposure prophylaxis.

## Notes

**Peer Reviewed**

**First Published Online:** November 22, 2024.
